# Patterns and predictors of place of cancer death for the oldest old

**DOI:** 10.1186/1472-684X-4-6

**Published:** 2005-10-08

**Authors:** Anna Lock, Irene Higginson

**Affiliations:** 1 Coventry Community Palliative Care Team, 25 Warwick Road, C/O Christchurch House, Grey Friars Lane, Coventry, CV1 2GQ, UK; 2 Department of Palliative Care and Policy, King's College London, Weston Education Centre, Cutcombe Road, Denmark Hill, London, SE5 9RJ, UK

## Abstract

**Background:**

Cancer patients increasingly are among older age groups, but to date little work has examined the trends in cancer among older people, particularly in relation to end of life care and death. This study describes the older population who die of cancer and the factors which may affect their place of death.

**Methods:**

A Cross-sectional analysis of national data was performed. The study included all people aged 75 and over dying of cancer in England and Wales between 1995 and 1999. The population was divided into exclusive 5 year age cohorts, up to 100 years and over. Descriptive analysis explored demographic characteristics, cancer type and place of death.

**Results:**

Between 1995 and 1999, 315,462 people aged 75 and over were registered as dying from cancer. The number who died increased each year slightly over the 5 year period (1.2%). In the 75–79 age group, 55 % were men, in those aged 100 and over this fell to 16%. On reaching their hundreds, the most common cause of death for men was malignancies of the genital organs; and for women it was breast cancer.

The most frequent place of death for women in their hundreds was the care home; for men it was hospitals. Those dying from lymphatic and haematopoietic malignancies were most likely to die in hospitals, those with head and neck malignancies in hospices and breast cancer patients in a care home.

**Conclusion:**

The finding of rising proportions of cancer deaths in institutions with increasing age suggests a need to ensure that appropriate high quality care is available to this growing section of the population.

## Background

Between 1991 and 2001 [1], the UK the population aged over 85 rose by 29.6%. Further since the 1950's the number of people aged over 100 has doubled every decade [2]. A shrinking wage earning group and the growing number of dependent older people is creating a society in which taxable income will fall, whilst demands on health and social services are already rising. This growing section of older people are often socially excluded and at risk of experiencing a combination of linked problems such as unemployment, poor skills, low incomes, poor housing, high crime environments, bad health and family breakdown [3]. Age-based discrimination can be experienced with poorer access to and availability of health care services [4,5].

Cancer is more common with increasing age [1] and the experience of having cancer in old age has been shown to be different to that of younger people [4]. Older people with cancer have multiple co-existent pathologies such as cardiac failure and chronic respiratory disease, prolonged recovery phase and rapid deterioration if the illness is left untreated [6]. Higher levels of functional dependence or co-morbidities[7] have been found in all ages to be associated with increased institutional death.

Meeting a person's choice regarding place of care and death at the end of life is seen as a useful outcome marker of the quality of palliative care. In younger age groups 50% to 80% of people wish to die at home. Increasing age in the general population [8] has been found to be related to preference for not dying at home, where as in Israel when home care patients were asked older people (50–59 yrs) were more likely to prefer death at home (95.2%) than the youngest (21–29: 50%)[9]. Little data is available on the preferences of the oldest old cancer patients or those who lack informal care givers [10]. Despite the critical position of choice of place of death in an older person's expression of their autonomy [11] older people have been found to recognise both practical and moral difficulties in achieving home death [12]. Expressing a preference for home death has been identified in the general population as a factor which may increase home deaths [13].

Previous data gathered on preference for place of death has often focused on home or 'elsewhere', very rarely has care home been reported as an option. An Australian interview survey of general population members found that 2.5% would prefer to die in a nursing home if terminally ill [8]. When patients referred to a palliative care hospital support team were asked, 3% stated that nursing home was their preferred place of terminal care [14].

Increasing age has been found to be associated with reducing the probability of dying at home or hospice and increasing death in care homes [7,9,13,15-19]. None of the studies specifically described the changes in place of death with increasing age beyond 84+.

The association of gender with place of death has been found in a number of studies. In general women are more likely to die in care homes[19], whilst men are generally more likely to die at home[15] or in hospital [18,20]. Italian data conflicts with this, in a region with no care homes, female home care patients were more likely to die at home than men[21]

The effect of cancer group on place of death has been found to be most pronounced for haematological malignancies. These have been repeatedly shown to be associated with death in hospitals [19,20,22]. This is unlike other tumour groups which have shown little consistency between studies.

The availability of caregivers has been highlighted as a factor which may influence place of death at any age of cancer patients. Having a spouse has been found to be associated with a reduced probability of death in a care home [7] and an increased number of care givers was also shown to increase the probability of home death [23-25].

However, no studies have analysed place of death in the oldest old. This study sought to examine the epidemiology of the oldest old dying with cancer and factors which may influence their place of death.

## Methods

Cross-sectional analysis was performed on routinely collected death registration data for cancer deaths (ICD 9 codes 140–239) in England and Wales, for the years 1995–1999, of all people aged 75 and upward under licence from the Office of National Statistics. Data included: age, gender, social class, country of birth, cause (ICD-9) and place of death.

Using SPSS for Windows v11.5, age was examined in 5 year exclusive cohorts ranging from those aged 75–79 years (the 'younger old') to those over 100 years and over (the 'oldest old'). Because of small numbers in the 105–110 cohort (n = 16), all those aged 100 years and over were analysed together. Trends in cause of death and country of birth were described for the whole population and separately by gender with increasing 5 year age cohorts.

## Results

A total of 315,462 of deaths from cancer of people aged 75 and over were registered in England and Wales. This accounted for 46% of all cancer deaths. There was an increase of 1.2% (783) between 1995 (62,266) and 1999 (63,049). In contrast the total number of cancer deaths fell by 5% between 1995 (140,791) and 1999 (133,749). Complete data was available regarding age, gender, cause of death and place of death. 99. 7% of social class information was missing.

**Figure 1:**
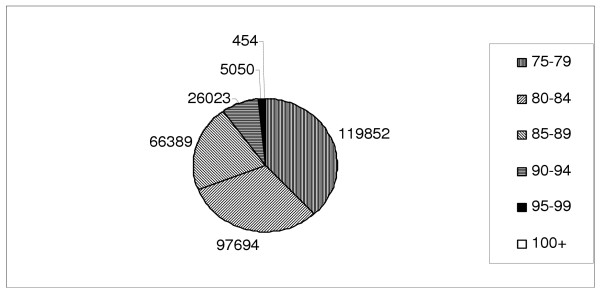
Pie chart of number of deaths by age cohort (N = 315,462).

**Figure 2:**
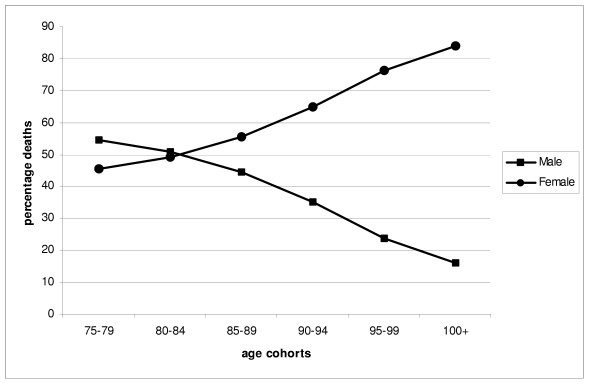
Graph of percentage deaths for men and women by age cohort (N = 315,462).

Of cancer deaths, 119,852 were aged from 75 to 79, and 454 were aged 100 and over (Figure 1). The oldest person registered as dying in this period was 108, 49% were men. The proportion of women rose from 45% of the 75–79 cohort to 84% of the 100 and over cohort (Figure 2).

Most people (92.8%, n = 292,795) had been born in England and Wales, the remainder comprised people from 148 other countries. Of those born outside of England and Wales, 21% came from the Irish Republic and 21% from Continental Europe. The proportion of those born outside of England and Wales fell towards the extreme of old age when compared to the younger old.

**Figure 3:**
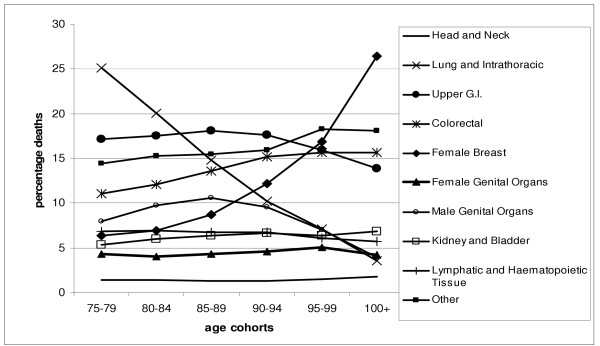
Percentage cause of cancer death by age cohort (N = 315,462).

For the population as a whole, the most common cause of death was lung and other intra-thoracic malignancies, which was recorded as the cause of death in 19.9% (n = 62,604) of deaths. In the 75–79 cohort, lung and other intra-thoracic malignancies comprised 25.1% of deaths – this fell to 3.5% in those over 100. In this oldest group the most common cause of death was female breast cancer, which contributed 26.4% of deaths, contrasting with 6.3% in the 75–79 cohort. The proportion dying of cancer described as 'other' rose from 14.5% in the 75–79 cohort to 18.1% in the over 100's (Figure 3).

When the population was described separately for men and women, the most common cause of death for men was lung cancer contributing 25.2% of all deaths and 29. 4% of those aged 75–79. On reaching their hundreds the most common cause of death for men were malignancies of the male genital organs (24.7%) with lung and other intra-thoracic malignancies contributing 11% of deaths.

For women, upper gastrointestinal malignancies were the most common cause of death overall, with 18.2% of total deaths. In the youngest cohort, 19.9% of deaths were due to lung and intra-thoracic malignancies. By the 80–84 cohort, gastrointestinal malignancies were the most common cause of death and remained so until the 94–99 cohort when breast cancer was the most common cause of death (22.2%), and reaching 31.5% in women over 100.

Most people died in hospitals (n = 156,334/50%), and fewest in hospices (n = 39,576/13%). Home deaths accounted for 19% of deaths, and care homes for the remaining 16% of deaths. There was a small increase in the percentage of deaths in hospices over time, from 11.5% in 1995, to 13.7% in 1999.

**Figure 4:**
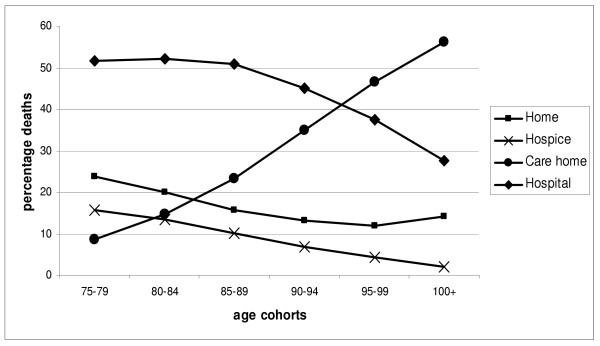
Percentage deaths in each place of death by age cohort (N = 307,613).

There were marked differences between the 'younger old' (aged 75–79 years) and the 'oldest' old (the over 100's) in place of death. Those dying in hospital reduced with increasing age, from 51.8% to 27.5%. Hospice deaths also fell, from 15.7% to 2%, as did home deaths, from 23.8% to 14.2%. Care home deaths increased from 8.7% in the 'younger old' to 56.3% of the 'oldest old' (Figure 4).

In the 'younger old', hospital was the most common place of death for both men and women. However, among the 'oldest old', care homes became the most common place of death (41.1%) for women. Although smaller in number, men in their 100's still died mainly in hospitals (49.4%). Care home deaths were the second most common place of death (29.2%).

The proportion of those dying in different settings varied with cause of death. Of those dying from malignancies of the lymphatic haematopoietic tissues, 66% died in general hospitals or multifunction site, compared to 45% for those with head and neck malignancies. Death in care homes was highest for those with breast cancer (31%), compared with 11% of those with lung and intra-thoracic malignancies. Hospice death was most common among those dying of head and neck tumours (19%), and least common among those with lymphatic and haematopoietic tissue malignancies (8%). Home was the most common place of death amongst those with upper GI malignancies (24%) closely followed by lung and intra-thoracic malignancies (23%).

For men and women within individual cancer types place of death was similar – although clearly breast cancer patients could not be compared. The highest frequency of death in care homes for men was cancer of the genital organs (19%) and, for women, was those dying of breast cancer (31%).

## Discussion

This study highlights the rich potential of an easily available, economic data source that has data for a whole population. This enabled a picture to be drawn of an often neglected population without encumbering individuals with interviews. As with all routine data analysis, the study was limited to variables which were previously collected and their completeness. Due to the cross-sectional study design, we were unable to control for confounding factors which may have influenced the results.

Nevertheless, our study demonstrates the increasing number of cancer deaths per year in the oldest old, contrasted with the overall fall of cancer deaths. The greater number of older women dying with cancer, is a reflection of the general population, which has disproportionately more women than men in extreme old age [2]. The low proportion of deaths of people born outside of England and Wales was unsurprising as these groups in the UK have a younger age profile than that of the white population[26], although this situation will change [27].

Our analysis of cause of death is likely to be limited by the accuracy of diagnosis and physician certification [28]. The older population are likely to have multiple pathologies and as a consequence it may be unclear as to the actual cause of death, demonstrated by the rising proportion of cause of cancer deaths described as 'other'. The lower levels of investigation in older people may mean in fact that their cancer is never diagnosed, leading to cases being missed from this study.

The finding that the most common cancer groups in the very old population were of breast and male genital organ origin is significant for service planners. Both diseases can have a protracted course, local and distant spread with associated complications, and multiple treatment options. It is likely that these tumour groups will become an increasing part of the cancer work load as our population ages. Therefore plans for longer term supportive and palliative care are needed[29].

Our finding of variations in place of death by diagnosis supports clinical experiences. Tumours which can lead to complex symptoms such as those of head and neck cancers can be especially difficult to palliate. These patients may be therefore more likely to be perceived to require specialist palliative care and be referred to hospices. The high proportion of deaths in hospitals for those dying of haematological malignancies has been previously documented in the general population[20] and may be linked to their health team having fewer links with specialist palliative care services and therefore reduced access to hospice beds and supportive services at home. It may also be related to the clinical course, which can be rapid with a very short period from diagnosis to death. Both of these factors can reduce the likelihood of home death[16].

Our data on place of death is limited by the data collection methods, in particular completion of the death certificate and coding variations. It only gives a snap shot of the dying pathway, and importantly does not tell us how long a person has been at that place before they died. Our findings support and build on earlier studies, showing a trend away from home death and towards care homes, with increasing age[7,9,18,30]. We found that this trend continues even to the oldest old. On a global scale there is wide range in the reported difference in place of death for the older population. In Italy 33% of those aged 75–84 died at home rising to 42.4% of those aged 85 and above[31]. In USA[20] and Australia[19] the picture seems to be similar to the UK, although this needs more investigation with direct comparative analysis.

The reasons for the variation in place of death as the population reaches the extremes of old age are complex. For many older people life in a care home is their reality. As by aged 65 and above 4% of people live in care homes, rising to 20% of people over 85 years old. Of all residents 75% are women [32]. So for a large number of older people who die in a care home, this is their de facto place of residence.

It is not possible using the data available to ascertain how long the older people recorded as dying in care homes had been resident there, in some cases care home deaths may be true home deaths. With hospices and hospitals increasingly discharging people for ongoing care to care homes the duration of admission may be as short as days, this will have implication for service planning achieved. However, the type of care needed will vary very much through a course of a palliative illness with the intensity of input required by carers increasing in the terminal phase.

Increasing in the UK, care home death may be in part due to the influence of General Practitioners who act as gate-keepers to services [33] and may perceive older people to have needs related to physical and social difficulties and be amenable to care homes [34] rather than having complex psychological and symptom control needs which may suggest referral to a specialist palliative care unit. It may be that the course of illness is less predictable, and not suitable for the relatively short length of hospice admission. The high proportion of care home and hospital deaths has implications for these organisations whose staff may not be trained to care for dying cancer patients. In many cases staff have a variable understanding of the concepts of safe amount of analgesia and definitions of euthanasia [34].

The previously noted reduction in proportion of patients preferring home as a place of death as death approaches[35] may be especially pertinent in the oldest dying. Increasing disability towards their life may be exacerbated by co-morbidities and lack of carers may make death at home less desirable. For stretched community health and social care services, which are often augmented by informal caregivers, it may not be possible to input enough care for older people who are more likely to be living alone. Previous data suggests that Hospice team involvement [16,21] and having special equipment [13] may increase the probability of home death. So for older people who are known to experience age-based discrimination with access to and availability of services [36] this may be another factor which reduces their probability of home death.

This highlights the issue of whether there can ever be a true choice. That realism and a desire to spare others the burden of care often clouds the issue of choice of place of care and death for a section of society that are already disadvantaged.

In the UK, there is a general trend towards increasing provision of care at home for people who require palliative care. This have been encouraged by studies that have shown that in general, people prefer care at home [10]. However, home death does not occur for the majority of older people, and so it is essential that appropriate care services are available in all settings, home, hospital and care home.

The death of a large proportion of the population in care homes and acute hospitals emphasises the need for all health care workers caring for older people in these settings to be adequately trained in generic palliative care in addition to training in complex needs assessment. Models for collaboration between primary and secondary care practitioners with palliative care specialists should be developed and implemented.

## Conclusion

46% of all cancer deaths occur in the 75 years and over population. The causes of cancer deaths changes with progression into extreme old age. Lung cancer becomes less common and breast and prostate cancer becomes the most frequent. Care homes and hospitals dominate the place of death in the oldest old, suggesting a need to target training and resources in these organizations as the general population ages.

## Competing interests

The author(s) declare that they have no competing interests.

## Authors' contributions

AL designed the study and performed the data analysis and interpretation as part of the MSc in Palliative Care at King's College London. IH acquired the data, conceived of the study idea, participated in its design, gave editorial advice and was the MSc supervisor. All authors read and approved the final manuscript.

## Pre-publication history

The pre-publication history for this paper can be accessed here:


